# Supplementary material for: High precision structured H∞ control of a piezoelectric nanopositioning platform
Source: PLoS One. 2023 Jun 16;18(6):e0286471. doi: 10.1371/journal.pone.0286471 (PMC10275421; doi:10.1371/journal.pone.0286471)
Supplement: Attachment — Submitted filename: Response to Reviewers.docx [file pone.0286471.s001.docx]

Response to editor:

1: This article has been modified to conform to PLOS ONE journal format.

2: The funding information has been deleted and modified in the Acknowledgements section.

3: Copies of Figures 12 and 13 have been uploaded, which are mentioned and described in the text, as shown in lines 419 and 422.

4:The data availability statement has been partially revised.

Response to reviewer 1:

Reviewer #1: The paper focuses on the combined problems of inherent resonant modes, low bandwidth and model uncertainty of the piezoelectric-driven nanopositioning platform. The system characteristics and performance requirements are analyzed and the structured H-infinity controller is designed to satisfy the multiple performance requirements. Although the paper is interesting, the following issues should be addressed:

1. The references should be updated, the literatures are a bit old. Also, the references review in the introduction is incomplete, which should be further improved.

**Response:** Some new references have been updated. In addition, to address the problem of incomplete review of references, this paper has been supplemented with a review of the feedforward/feedback combined control method, see lines 68-74 marked in red.

2. Since the paper first models the piezoelectric positioning system by considering the hysteresis effect, it may be better to discuss the classical hysteresis models, such as the Preisach model, Bouc-Wen model, Duhem model, Maxwell model, etc. In addition, some advanced neural network based hysteresis models should also be discussed, such as the gated recurrent unit based hysteresis model, improved neural Turing machine based hysteresis model, etc. The additional discussion can make readers understand the reason of adopting the modeling method in the paper.

**Response:** For classical hysteresis models such as physics-based models, phenomenological models including differential equation-based hysteresis models (common Duhem, Backlash-like and Bouc-Wen models), operator-based hysteresis models (typical Preisach models) and other hysteresis models (based on artificial neural networks, fuzzy systems and support vector machines, etc.) intelligent models), etc., which have been added to the discussion in the models section of this paper. See lines 164-181 marked in red.

3. Apart from the open-loop Bode diagram, it may be better to provide the input-output curves of the system to present the modeling performance more intuitively.

**Response:** The discussion in this paper focuses mainly on control methods, and due to the lack of a real experimental platform, its control object model is the model after system identification borrowed from reference [40], which has been marked in this paper with citations and specific details available in reference [40]. See lines 184-187 marked in red.

4. The author should pay attention to grammar mistakes, and the paper needs careful proof-reading.

**Response:** The paper has been corrected for multiple grammatical errors and has been carefully proofread.

Response to reviewer 2:

Reviewer #2: This research is quite interesting by incorporating the newly proposed structured H-inf control into the nano-positioning control. As can be learnt, the order of the proposed controller is low for easy implementation. And another advantage is that the functions of each parts are explicable, which outperforms the conventional H-inf controller. Despite the advantages presented in this paper, there are some aspects that might help improve the quality and readability, listed as follows:

1. I'm curious about the high frequency interference signal, what's it? Sinusoidal signals or random signals?

**Response:** External disturbance interference signal may be any form of high frequency signal. The high-frequency disturbance signal test subsection of this paper is selected as a sinusoidal signal close to the resonant frequency, respectively, $u_{d}=0.2\sin(139t)$ and $u_{d}=0.2\sin(205t)$. The original text was omitted in the editing and has been revised, see lines 416-417 and lines 422-423 in red.

2. For the choice of W2, eq(18), is it able to explain the way to choose W2? To be more specific, among several alternatives, how can one decide which is the best? What are the expected results on choosing different W2's? BTW: In fig5, zero-load bode diagram is expected as well.

**Response:** (1): The original paper is indeed vague about the selection of W2, which has been explained and modified in the corresponding part of the paper, see lines 303-308 in red. The multiplicative uncertainty at 600g and 1000g load is calculated by equation (13), while for all the uncertainties, the appropriate weighting function W2 is designed. For all frequency bands, so that the multiplicative uncertainty $\Delta$ needs to satisfy$\left\|\Delta\right\|\leq\left\|\Delta_{m}\cdot W_{2}\right\|$, where $\Delta_{m}$ is the unitized regression, the selection of W2 can satisfy the above conditions.

(2): There is no best W2, and the expected result of choosing W2 is that the solved control system has robust stability, that is, the optimal parameters of the controller are obtained after satisfying $\left\|W_{2}\left(s\right)T_{2}\left(s\right)\right\|_{\infty}\leq\gamma$ , which is explained in the subsection on control performance analysis.

(3) Fig.5 shows the weighting function and the multiplicative regression diagram under load. The curves corresponding to 600 and 1000g are the multiplicative uncertainties under 600 and 1000g load calculated by equation (13), respectively, which is not a zero-load bode diagram and can be compared with Fig.2 or different. The original description of Fig. 5 is indeed misleading and has been revised.

3. In the introduction, when the structured H-inf integrated control is firstly mentioned at line 78, it should be more comprehensive to introduce the essence of the "structured H-inf control". Or in other words, briefly explain "what is a structured H-inf control".

**Response:** The essence of the structured H∞ control strategy has been added in that section. The core of structured *H*_∞_ control theory is a locally optimal control strategy that balances control performance and controller complexity. Firstly, the reasonable controller structure is designed according to the system characteristics and control objectives. Secondly, the "standard H∞ parametric matrix" with multiple performance decoupled outputs and multiple controllers mixed and nested is derived to build a standard structured integrated control form. Finally, the reasonable weighting function is designed to solve for the optimal controller parameters. See lines 84-90 marked in red.

4. The plots should be carefully redesigned, for example:
a) the text in the figure is small for printed versions.
b) the figures is not kind for color-blind people, and is not distinguishable in gray-printed version.

**Response:** a) The proposed figure has been modified for the part of the figure with smaller text, such as Figure 5, Figure 6, Figure 7, Figure 8, Figure 9 in (a), (b), (c) , Figure 11, Figure 12 and Figure 13.

b) In response to the data on the bar chart in Figure 10, which did appear to be unfriendly, the data have been removed and modified, and the values can be viewed in Table 1. Some color charts were used because the journal article was published in color upon review, and will be revised subsequently if there are any problems.
