# Supplementary material for: High precision structured H∞ control of a piezoelectric nanopositioning platform
Source: PLoS One. 2023 Jun 16;18(6):e0286471. doi: 10.1371/journal.pone.0286471 (PMC10275421; doi:10.1371/journal.pone.0286471)
Supplement: Attachment — Submitted filename: Response to Reviewers.docx [file pone.0286471.s002.docx]

Response to reviewer 2:

1. The place of the newly added content discussing the massive piezoelectric models is not suitable. It might be better to place it right at the beginning of the Section Model, rather than the middle.

**Response:** Added discussion of the model has been placed at the beginning of the model introduction

2. Section name "Experimental comparative analysis" is misleading since there is no on-site experiments, e.g., only simulation is provided. It should be more informational.

**Response:** The chapter title " Experimental Comparative Analysis" has been changed to "Simulation Comparative Analysis"

3. Some more words on the plots:
a) For font size: The font size for the x/y-labels can be further enlarged. These label font size being much smaller than the normal font is not suggested unless there are adequate reasons. This rule applies to the legends as well.
b) For colors: Not all people prefer e-papers, some reads the printed version. So, it is suggested that the colors are chosen that they are distinguishable in gray-printed version. For example, distinguish them with different line styles (dashed, dotted, dash-dotted, solid). Proofreading the paper one a gray-printed one is a good habit.
c) For data values: In fig 10, the data values help comparison and should be kept. The modifications suggested are:
1) Adjust the text size;
2) Differ the bars with different patterns (different colors are welcomed at the same time);
3) Keep the data value at the top of the bars, in black.

**Response:**
1) The text in the diagram has been resized.

2) The coloured lines have been distinguished by different line styles, which are also distinguishable in the grey printed version, see Figs. 2, 5, 6, 7, 8, 9, 11, 12 and 13 for the modified images.

3) Figure 10 has been modified to retain the data values at the top of the bars and the bars are differentiated by a different style.
